# Association Involving Systemic Arterial Hypertension, Tinnitus, and Hearing Loss: A STROBE-Based Review

**DOI:** 10.1055/s-0046-1818563

**Published:** 2026-04-30

**Authors:** Luíza Arouck Tavares, Rhuan Peres, Lucas Brabo Redig, Ana Clara Reis Guilhon, Lorenzo Giordano do Couto, Brenda Nazaré Andriolo

**Affiliations:** 1 Department of Medicine, Centro Universitário do Estado do Pará (CESUPA), Belém, PA, Brazil

**Keywords:** research design, hypertension, otorhinolaryngology

## Abstract

**Introduction:**

Systemic arterial hypertension (SAH) is a multifactorial condition that has the potential to affect inner ear function, thereby leading to sensorineural hearing loss and tinnitus. As the population ages, there is an increasing prevalence of sudden auditory impairment and auditory disorders. Therefore, it is essential to investigate this connection. are

**Objective:**

To estimate the association between sudden acoustic hearing loss (SAH) and tinnitus/hearing loss, and to evaluate the methodological quality of related research using the Strengthening the Reporting of Observational Studies in Epidemiology (STROBE) criteria.

**Methods:**

We employed an analytical cross-sectional design, with a review of observational studies published until April 2025 in English, Portuguese, or Spanish. The evaluation of articles was conducted using the STROBE checklist, and the studies were categorized as *adequate* , *inadequate* , or *insufficient* .

**Results:**

The prevalence of hearing impairment presented a significant correlation with hypertension (57.33%), followed by tinnitus (56.3%). Most of the topics were classified as *adequate* , even though the “Results” sections of the articles presented 12% of inadequacy, and the “Additional Information” sections demonstrated the most insufficiency (80%). Furthermore, the “Discussion” sections revealed a notable concern, with 12.5% of the respondents expressing dissatisfaction.

**Conclusion:**

A substantial degree of heterogeneity was observed across studies, underscoring the necessity for standardized future research to facilitate more reliable comparisons.

## Introduction

Systemic arterial hypertension (SAH) is a multifactorial condition characterized by the sustained elevation of blood pressure. Despite its classification as a silent disease, some patients report symptoms such as headaches, dizziness, and tinnitus. 1 In such cases, patients may present with systemic disorders, including target-organ damage, which may involve the auditory system. 2 As the population ages, the prevalence of chronic diseases, including SAH and inner-ear disorders, rises. 3 Consequently, the investigation of possible associations involving these factors becomes pertinent.

Hearing loss is defined as a reduction in the ability to perceive sound. It can be classified into different types, including sensorineural hearing loss, which results from alterations in the inner ear or central auditory pathways. 4 Meanwhile, tinnitus is defined as the perception of sound in the absence of an external auditory stimulus. This condition may manifest as a continuous or intermittent noise that significantly impacts the quality of life of the affected individuals. 5

Research 6 7 indicates that diseases affecting the circulatory system may have implications for inner ear function, given the dependence of auditory sensory cells on a sufficient supply of oxygen and nutrients for optimal functionality. The cochlear microcirculation is supplied by the common cochlear branch of the labyrinthine artery, with no collateral or alternative flow. This anatomical configuration renders the cochlea particularly vulnerable to vascular changes, such as those observed in SAH. 6 Therefore, there is a body of research 7 that suggests a potential association between SAH and auditory dysfunction, particularly in elderly individuals.

In addition to potential alterations in microcirculation, other factors have been subjects of debate, including oxidative stress induced by hypertension and the effects of antihypertensive medications on hearing. 8 Certain medications, including diuretics, calcium channel blockers (CCBs), and angiotensin-converting enzyme inhibitors (ACEIs), possess ototoxic properties and have the potential to contribute to the development of hearing loss or tinnitus. 1 However, the relationship involving SAH, hearing loss, and tinnitus has not been fully elucidated, requiring further investigation to differentiate correlation from causality and to comprehend the underlying pathophysiological mechanisms.

Hearing loss has been demonstrated 9 to have a considerable impact on communication, potentially resulting in social isolation, diminished quality of life, and an elevated risk of depression and cognitive decline. Tinnitus can result in significant discomfort and psychological distress. 10 Therefore, ascertaining the modifiable risk factors is imperative for the prevention and management of these conditions.

Given the heterogeneity of findings and methodologies employed in the literature, it is imperative to synthesize the results of studies and to critically assess their methodological quality. The objectives of the current review are to provide an overview of the association between systemic arterial hypertension and auditory symptoms, and to highlight the strengths and weaknesses of the current evidence. In order to accomplish these objectives, the Strengthening the Reporting of Observational Studies in Epidemiology (STROBE) protocol was applied to cross-sectional studies. The results of the current review will guide future research and clinical decision making.

## Methods

The present is an analytical, cross-sectional research conducted from March 2025 to April 2025, analyzing previously-published studies with prior approval from an ethics committee, available on the PubMed, which associate SAH with otological complications, specifically tinnitus and/or hearing loss. Thus, submission of the current study to an ethics committee was not required, as no individuals underwent any new intervention.

A sensitive search strategy was adopted to conduct the research, combining official terms and synonyms on this topic. The searches were performed using the following descriptors: (( *Hypertension* [Mesh]) AND ( *Tinnitus* [Mesh])); (( *Hearing Loss, Sensorineural* [Mesh]) AND ( *Hypertension* [Mesh])), which were combined and filtered for human studies. The studies were selected for inclusion based on an independent review of titles and abstracts. Observational cross-sectional studies, written in any language, submitted since the creation of the database up to April 2025, were included, while studies that were inaccessible due to financial or logistical constraints were excluded.

The selected articles underwent a rigorous quality assessment according to the STROBE criteria, which consist of a set of recommendations aimed at improving the quality of reports on observational cross-sectional studies. 11

Widely adopted by authors and editors to assess the quality of scientific work, STROBE comprises a checklist of 22 items, designed to recommend essential topics in each section (title, abstract, introduction, methodology, results, discussion, and funding) of a study covered by this framework. This instrument aims to contribute to a more rigorous writing process for these studies, making it easier to select those that will guide decision making in healthcare practice. 12

Thus, six topics related to the study sections (title/abstract, introduction, methods, results, discussion, and additional information) were evaluated, totaling 23 subtopics. After reading each study, they were classified as follows: 1) adequate – if it met all or most of the STROBE's requirements for that section; 2) inadequate – if it did not meet the STROBE's requirements for that section; and 3) insufficient – if the article lacked sufficient information for an adequate analysis of that section, according to the STROBE statement. The data collected from each article were tabulated and analyzed in digital spreadsheets using the Microsoft Excel 2016 (Microsoft Corp.) software.

## Results

**Fig. 1:**
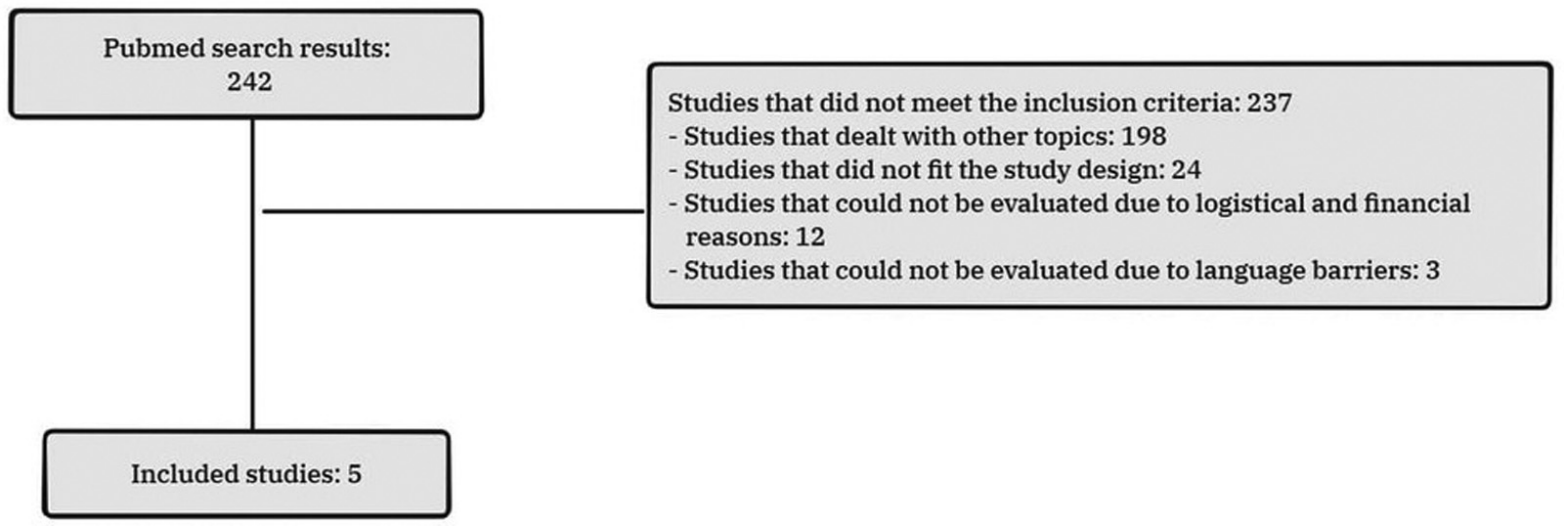
Flowchart of screening, inclusion, exclusion, and selection of studies.

Of the total 242 articles analyzed, 237 did not meet the proposed inclusion and exclusion criteria. Therefore, 5 articles 13 14 15 16 17 were reviewed in the present study ( Fig. 1 ). The studies were conducted in Brazil, 13 14 16 China, 17 and Canada, 15 with the primary sample population consisting of patients with SAH. In this context, tinnitus was the otological complication most associated with arterial hypertension (56.3%), followed by high-frequency hearing loss (44.3%) ( Table 1 ).

**Table 1:** Characterization of the analyzed articles

Author	Country	Sample	Target population	Otologic complications	Association: n (%)
Samelli et al. 16 (2021)	Brazil	900 patients	General	● Tinnitus ● MLFHL ● HFHL ● Hearing loss at any frequency	– 137 (45.8%); – 39 (13%); – 133 (44.3%); – 172 (57,33%)
Carneiro et al. 13 (2022)	Brazil	473 patients	General ≥ 18 years	● Tinnitus	– 117 (56.3%)
Gibrin et al. 14 (2013)	Brazil	498 patients	General ≥ 60 years	● Tinnitus RE ● Tinnitus LE	– 79 (41.36%); – 54 (28,27%)
Ramage-Morin et al. 15 (2021)	Canada	6,198 patients	General > 18 and < 80 years	● Only tinnitus ● Only hearing loss ● Tinnitus and hearing loss	– 1.143 (33.5%); – 1.401 (38,18%); – 891 (28.20%)
Zhang et al. 17 (2023)	China	242,811 patients	General ≥ 18 and ≤ 60 years	● HFHL	– 4.409 (13,69%)

**Abbreviations:** HFHL, high-frequency hearing loss; LE, left ear; MLFHL, medium- and low-frequency hearing loss; RE, right ear.

Regarding the evaluation of the articles based on the STROBE criteria, we observed that all topics were predominantly classified as *adequate* . Among them, the “Introduction” and “Title and Abstract” demonstrated the best performance, both with a percentage of 100%. Regarding inadequacy, the “Results” sections presented the highest percentage (12%). Conversely, regarding the *insufficient* classification, the highest percentage was found in “Additional Information” (80%), followed by “Discussion” (12.5%) ( Table 2 ).

**Table 2:** Classification of the articles according to the 6 topics categorized by the STROBE statement

Classification	Title and abstract (STROBE topic 1)	Introduction (STROBE topics 2–3)	Materials and methods (STROBE topics 4–12)	Results (STROBE topics 13–17)	Discussion (STROBE topics 18–21)	Additional Information (STROBE topic 22)
Adequate	10 (100.00%)	10 (100.00%)	40 (88.88%)	21 (84%)	17 (85%)	1 (20%)
Inadequate	0 (0.00%)	0 (0.00%)	0 (0.00%)	3 (12%)	0 (0.00%)	0 (00.00%)
Insufficient	0 (0.00%)	0 (0.00%)	5 (11.11%)	1 (4%)	3 (15%)	4 (80%)
**TOTAL**	**12** **(100.00%)**	**12** **(100.00%)**	**45** **(100.00)**	**25** **(100.00%)**	**20 (100.00%)**	**5** **(100.00%)**

**Abbreviation:** Strengthening the Reporting of Observational Studies in Epidemiology (STROBE).

The analysis of the subtopics revealed that “Informative and Balanced Abstract,” “Context/Justification (Introduction),” “Objectives (Introduction),” “Study Design (Materials and Methods),” “Participants (Materials and Methods),” and “Outcome (Results)” were classified as *adequate* in all evaluated articles. The subtopics “Bias (Materials and Methods),” “Generalization (Discussion),” and “Additional Information” had the highest percentages in the *insufficient* classification ( Table 3 ).

**Table 3:** Classification of the articles according to the topics of the STROBE statement

STROBE subtopics/classification (n = 5 articles)	Adequate: n (%)	Inadequate: n (%)	Insufficient: n (%)
1a – Study design in the title or abstract	5 (100.00%)	0 (0.00%)	0 (0.00%)
1b – Informative and balanced abstract	5 (100.00%)	0 (0.00%)	0 (0.00%)
2–Background/Rationale (Introduction)	5 (100.00%)	0 (0.00%)	0 (0.00%)
3–Objectives (Introduction)	5 (100.00%)	0 (0.00%)	0 (0.00%)
4–Study design (Materials and Methods)	5 (100.00%)	0 (0.00%)	0 (0.00%)
5–Setting (Materials and Methods )	5 (100.00%)	0 (0.00%)	0 (0.00%)
6–Participants (Materials and methods)	5 (100.00%)	0 (0.00%)	0 (0.00%)
7–Variables (materials and Methods)	5 (100.00%)	0 (0.00%)	0 (0.00%)
8–Data sources/measurement (Materials and Methods)	5 (100.00%)	0 (0.00%)	0 (0.00%)
9–Bias (Materials and Methods)	0 (0.00%)	0 (0.00%)	5 (100.00%)
10–Study size (Materials and Methods)	5 (100.00%)	0 (0.00%)	0 (0.00%)
11–Quantitative variables (Materials and Methods)	5 (100.00%)	0 (0.00%)	0 (0.00%)
12–Statistical methods (Materials and Methods)	5 (100.00%)	0 (0.00%)	0 (0.00%)
13–Participants (Results)	4 (80.00%)	1 (20.00%)	0 (0.00%)
14–Descriptive data (Results)	4 (80.00%)	0 (0.00%)	1 (20%)
15–Outcome data (Results)	5 (100.00%)	0 (0.00%)	0 (0.00%)
16–Main results (Results)	3 (60.00%)	1 (20.00%)	1 (20.00%)
17–Other analyses (Results)	4 (80.00%)	1 (20%)	0 (0.00%)
18–Key results (Discussion)	5 (100.00%)	0 (0.00%)	0 (0.00%)
19–Limitations (Discussion)	4 (80.00%)	0 (0.00%)	1 (20.00%)
20–Interpretation (Discussion)	5 (100.00%)	0 (0.00%)	0 (0.00%)
21–Generalizability (Discussion)	2 (40.00%)	0 (0.00%)	3 (60.00%)
22–Funding (Additional information)	1 (20.00%)	0 (0.00%)	4 (80.00%)
**TOTAL**	**95 (84.07%)**	**3 (2.65%)**	**15 (113.27%)**

**Abbreviation:** Strengthening the Reporting of Observational Studies in Epidemiology (STROBE).

## Discussion

The current study presents a comprehensive mapping of the association between systemic arterial hypertension (SAH) and its primary otological complications, outlining the variability of findings among the analyzed studies. 13 14 15 16 17 It also identifies important methodological limitations and potential biases that may affect the reliability of the results. Collectively, the evidence suggests a variable yet consistent association involving SAH, hearing loss, and tinnitus.

To the best of our knowledge, the current study constitutes the first mapping conducted to analyze studies associating arterial hypertension and its two main otological complications. This mapping provides a panoramic view of the disorders addressed and the research conducted on the subject, and it also enables a qualitative categorization of these studies using an approach based on the STROBE statement.

We found that the studies selected 13 14 15 16 17 presented certain discrepancies in their findings. The associations involving SAH and tinnitus and/or hearing loss found in the analyzed studies are highlighted in Table 1 , and they seem heterogeneous, with a high occurrence of hearing loss, showing association rates of 57.33% 16 and 38.18%. 15 At the same time, lower association rates were observed, such as 13.69%. 17 This variation may be explained by the different sample sizes of the studies (900 16 and 6,198 15 compared to 242,811 17 patients) and the different population profiles in the three countries where these studies were conducted (Brazil, 16 Canada, 15 and China 17 ).

Another important factor to consider is the diversity of diagnostic methods used to determine hearing loss and the subjective nature of tinnitus diagnosis. With various tests available, it is essential to associate data from medical history, symptoms, and physical examination findings for better characterization, which hinders standardization in diagnosis.

It is also worth noting that all of the studies 13 14 15 16 17 analyzed were conducted with patient populations that overlap. This may explain the lack of additional discrepancies and the limited heterogeneity among the obtained data. Furthermore, there is a significant discrepancy in the sample sizes of the studied populations, ranging from a minimum of 473 to a maximum of 242,811 17 participants. Additionally, the patient profiles were similar across the studies in important epidemiological aspects, mostly encompassing the adult population of both sexes.

A possible bias of the present study lies in the fact that the patients examined in the analyzed articles were entirely from tertiary care centers. Since tertiary centers are responsible for managing and treating specific conditions, there may be a greater association with the evaluated complications compared to population-based studies in large communities, in which patients may not have a history of follow-up in such centers, potentially leading to lower association rates.

Finally, the lack of methodological quality, assessed according to the STROBE statement, was a persistent issue identified by the researchers. Due to this, many articles could not be analyzed, as their association rates were omitted or incorrectly/inaccurately reported, preventing proper comparison and sometimes leading to over- or underestimation of certain results.

## STROBE

The topics of the STROBE statement that were most adequately addressed in most of the analyzed studies were “Title/Abstract” and “Introduction.” This can be attributed to the simplicity required for writing these sections, which do not demand extensive technical knowledge or a broad theoretical understanding of the study methodology. Furthermore, the first two topics had more than one subtopic, and fulfilling the requirements of just one of them was enough to consider the topic *adequate* .

The “Results” section was the one that showed the highest level of inadequacy in the evaluation, reflecting the difficulty most authors have in grounding their research in what are fundamental points for a good understanding by the reader and the relevance of the study within the scientific community. Poorly-elaborated results hinder the understanding of the research findings, making it impossible to use them as references for other works, such as the current.

In this context, in the “Materials and Methods” section, the subtopic “Bias” was the one that was most often rated as *insufficient* , showing the challenge in precisely identifying the factors that can be characterized as such. Studies with bias produce results that systematically differ from the truth. It is important for the reader to know what measures were taken during the study to reduce the potential and risk of bias occurrence. As for the results, the most negatively-rated subtopic was “Main Results.” Authors should explain all potential confounding factors considered and the criteria for including or excluding variables in statistical models. Decisions about variable inclusion or exclusion should be guided by knowledge or explicit assumptions to validate the results and facilitate the reader's understanding.

Regarding the “Discussion” section, the subtopic that was most inadequate in the evaluation was “Generalization,” which refers to the discussion of the external validity of the results. We found that, in half of the studies, this approach was missing, directly affecting how the data is interpreted by the reader, who may incorrectly generalize a specific result and form an erroneous understanding of the studied topic.

“Additional Information,” with the subtopic “Funding,” was found to be insufficient in most projects. This indicates that the authors did not prioritize the financial aspects of their studies, failing to be transparent with the readers, making it difficult to infer the study's reproducibility and preventing the testing of its feasibility. Furthermore, there was a lack of information about any potential sources of bias related to the funding of the work.

A limitation to the present study is the fact that the search was only conducted in one database (PubMed), restricting the range of analyzed studies to a subset of the total articles produced on this topic. Additionally, it widely covered the association involving SAH and tinnitus or hearing loss, resulting in research that did not necessarily follow the same reasoning, making a direct comparison of the associations impossible.

## Conclusion

The association involving SAH and its otologic complications, particularly hearing loss and tinnitus, seems legitimate but variable, influenced by comorbidities, external factors, and methodological differences among studies. The lack of standardization and frequent methodological shortcomings, as assessed by the STROBE protocol, highlight the need for more rigorous, standardized future research to ensure greater reliability and comparability of findings.
